# Mechanical Force Triggers Macrophage Pyroptosis and Sterile Inflammation by Disrupting Cellular Energy Metabolism

**DOI:** 10.3390/ijms26073321

**Published:** 2025-04-02

**Authors:** Hao Tan, Guoyin Yang, Ye Zhu, Xinyi He, Lan Yang, Yun Hu, Leilei Zheng

**Affiliations:** 1 College of Stomatology, Chongqing Medical University, Chongqing 401147, China; 18375761068@163.com (H.T.); 2022120706@stu.cqmu.edu.cn (G.Y.); zhuye0617@outlook.com (Y.Z.); 2020121237@stu.cqmu.edu.cn (X.H.); 2021440057@stu.cqmu.edu.cn (L.Y.); 2 Chongqing Key Laboratory of Oral Diseases and Biomedical Sciences, Chongqing 401147, China; 3 Chongqing Municipal Key Laboratory of Oral Biomedical Engineering of Higher Education, Chongqing 401147, China

**Keywords:** mechanical force, macrophage, pyroptosis, sterile inflammation, energy metabolism, orthodontic tooth movement

## Abstract

Mechanical force regulates tissue remodeling during orthodontic tooth movement (OTM) by inducing macrophage-mediated sterile inflammatory responses. Pyroptosis, as an inflammatory form of programmed cell death, triggers a robust inflammatory cascade by activating the inflammasome. Although recent reports have demonstrated that pyroptosis can be activated by mechanical force, it remains unclear whether and how orthodontic force induces macrophage pyroptosis and sterile inflammation. In this study, by establishing a rat OTM model and a force-loaded macrophage model, we found that force induces Caspase1-dependent pyroptosis in macrophages and activates sterile inflammation both in vivo and in vitro. Mechanistically, we uncovered that mechanical force disrupts macrophage energy metabolism, characterized by an imbalance between lactate dehydrogenase A (LDHA) and pyruvate dehydrogenase (PDH), as well as mitochondrial dysfunction. Notably, inhibiting pyruvate dehydrogenase kinase 1 (PDK1) effectively restored this metabolic balance, thereby alleviating pyroptosis and sterile inflammation in force-stimulated macrophages. Overall, this study elucidates that force induces macrophage pyroptosis and sterile inflammation, and further identifies imbalances in the LDHA/PDH ratio and mitochondrial dysfunction as pivotal mechanistic features. These insights offer novel perspectives and potential therapeutic targets for the precise and effective modulation of OTM.

## 1. Introduction

Orthodontic tooth movement (OTM) is a complex biomechanical process involving the movement of teeth within the alveolar bone, driven by mechanical force applied to the teeth [1]. These forces not only cause physical tooth movement but also trigger a series of biological responses, among which force-induced sterile inflammatory responses are particularly critical [2]. Characterized by the recruitment of immune cells and the release of cytokines, sterile inflammation is an inflammatory response caused by mechanical force or other non-infectious stimuli in the absence of pathogen infection [3]. It plays a crucial regulatory role in the initial tissue destruction and subsequent tissue remodeling during OTM [4]. Macrophages, as key innate immune cells in periodontal tissues, respond to mechanical force stimulation by secreting various inflammatory factors, such as TNF-α, IL-1β, and IL-6, which modulate local sterile inflammation during OTM [4,5].

While the role and significance of sterile inflammation in OTM have gradually been decrypted, the challenge of efficiently controlling this inflammation within a safe range remains to be addressed. Sterile inflammation can act as both a friend and a foe in orthodontic treatment, depending on its intensity and duration [6]. Transient and mild inflammation is generally considered beneficial for tooth movement and alveolar bone remodeling, whereas persistent and excessive inflammation can lead to adverse outcomes, including tissue damage and root resorption [1]. From an immunological standpoint, the immune response during OTM progresses through three distinct phases—initial, lag, and linear—each characterized by the activation, resolution, and stabilization of sterile inflammation, respectively [4]. During the initial phase, monocytes and macrophages infiltrate the affected tissues and release inflammatory factors. In the lag phase, the influx of inflammatory cells ceases, and macrophages begin to clear necrotic debris. Finally, in the linear phase, immune cells secrete anti-inflammatory factors to help restore tissue homeostasis. Therefore, identifying effective immune targets to modulate the sterile inflammatory response in a manner that enhances efficiency and ensures safety may hold the key to optimizing orthodontic treatment outcomes.

Pyroptosis, a form of programmed cell death activated by inflammasomes, is intricately linked to the development of sterile inflammation [7]. In the canonical pathway, the Nod-like receptor protein 3 (NLRP3) inflammasome activates Caspase-1, which subsequently cleaves Gasdermin D (GSDMD) and pro-IL-1β into their active forms, N-GSDMD and mature IL-1β [8]. This process leads to the formation of pores in the cell membrane mediated by N-GSDMD, through which cellular contents, including IL-1β, are released into the extracellular space, thereby triggering a robust inflammatory response [9]. Recent studies have explored the effects of mechanical force on pyroptosis in periodontal ligament cells (PDLCs), finding that mechanical compressive stress induces Caspase-1-dependent pyroptosis in PDLCs [10]. Additionally, NLRP3 inflammasome activation has been suggested to contribute to cyclic stretching-induced pyroptosis and IL-1β release in human periodontal ligament cells [11]. Despite these findings, research on the link between mechanical force and macrophage pyroptosis in the context of OTM remains limited. It remains unclear whether and how orthodontic force regulates sterile inflammation by inducing macrophage pyroptosis, and the underlying mechanisms require further elucidation.

The cellular energy metabolic state has been confirmed to be closely related to pyroptosis and associated inflammation [12]. Cellular energy metabolism mainly occurs through two pathways: glycolysis and oxidative phosphorylation, with mitochondria playing a central role [13,14,15]. Studies have shown that downregulation of glycolysis in alveolar macrophages can significantly improve cell pyroptosis and airway inflammation [16]. Other reports have indicated that inhibiting pyruvate dehydrogenase kinase (PDK) to enhance macrophage aerobic respiration can attenuate NLRP3 inflammasome activation and pyroptosis [17]. Moreover, dysfunctional mitochondria have been proven to activate the NLRP3 inflammasome and pyroptosis by releasing mitochondrial reactive oxygen species (mtROS) [18,19]. However, it is still unclear whether mechanical force can induce similar energy metabolic changes in macrophages to those reported in the aforementioned studies, thereby triggering pyroptosis and sterile inflammation.

Based on the above background, this study aims to elucidate whether and how mechanical force induces pyroptosis and sterile inflammation in macrophages. Using an OTM rat model and an in vitro macrophage mechanical loading model, we found that mechanical force triggers Caspase-1-dependent pyroptosis in macrophages and activates sterile inflammation by disrupting energy metabolism. Furthermore, we established the key role of PDK1 in this process. This study reveals a novel mechanism by which mechanical force activates macrophage pyroptosis and sterile inflammation through the energy metabolism pathway, providing a new theoretical basis and potential intervention targets for inflammation management in OTM.

## 2. Results

### 2.1. Force Triggers Sterile Inflammation During the Bone Remodeling Process of OTM

**Figure 1:**
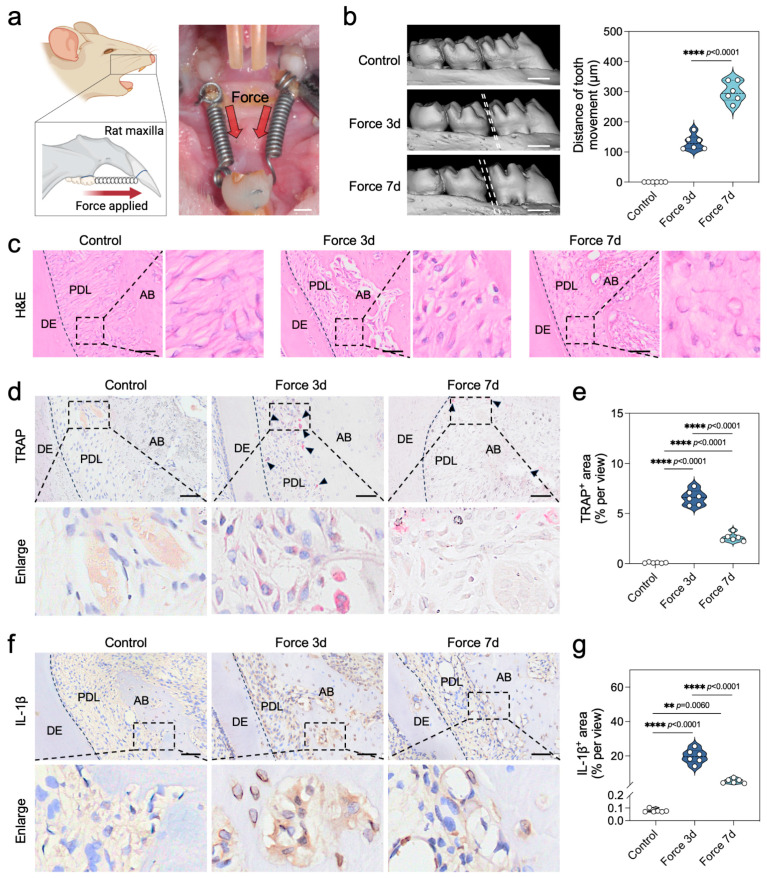
Force triggers sterile inflammation during the bone remodeling process of OTM. (**a**) Schematic and photographic images of the rat OTM model construction. Scale bar = 2000 μm. (**b**) Micro-CT scanning and reconstruction images of the maxilla in OTM rats and analysis of tooth movement distance (*n* = 6). Scale bar = 1000 μm. (**c**) H&E staining images of tissues on the pressure side of the first molar in OTM rats. Scale bar = 200 μm. (**d**,**e**) TRAP staining images of tissues on the pressure side of the first molar in OTM rats and analysis of the proportion of TRAP-positive areas (*n* = 6). Black arrows indicate TRAP-positive areas. Scale bar = 200 μm. (**f**,**g**) Immunohistochemical staining of IL-1β and analysis of the proportion of IL-1β-positive areas in tissues on the pressure side of the first molar in OTM rats (*n* = 6). Scale bar = 50 μm. ** *p* < 0.01; **** *p* < 0.0001. DE: Dentin, PDL: periodontal ligament, AB: alveolar bone.

To investigate the level of sterile inflammation during the bone remodeling process of OTM, we established a canonical force-induced rat OTM model, as depicted in Figure 1a. Micro-CT imaging provided compelling evidence of model efficacy, with the OTM distance in rats incrementally advancing to (132.31 ± 24.71) μm and (299.61 ± 33.86) μm at the 3-day and 7-day marks, respectively, post-force application (Figure 1b). Hematoxylin and Eosin (H&E) staining corroborated the absence of aberrant tooth root resorption attributable to the applied force (Figure 1c). Our findings from tartrate-resistant acid phosphatase (TRAP) staining underscored a pronounced surge in the quantity of TRAP^+^ osteoclasts on the compression side of periodontal tissues within the initial 3 days of force application, which subsequently waned by the 7-day interval (Figure 1d,e). Paralleling these observations, immunohistochemical assessment of the inflammatory biomarker IL-1β showed that the expression of IL-1β^+^ positivity followed a similar trend to that of TRAP^+^ osteoclasts (Figure 1f,g). Collectively, these data robustly demonstrate the activation of sterile inflammation by force during the bone remodeling cascade of OTM, with peak inflammatory responses detectable at the 3-day juncture.

### 2.2. Force Induces Macrophage Pyroptosis During OTM

**Figure 2:**
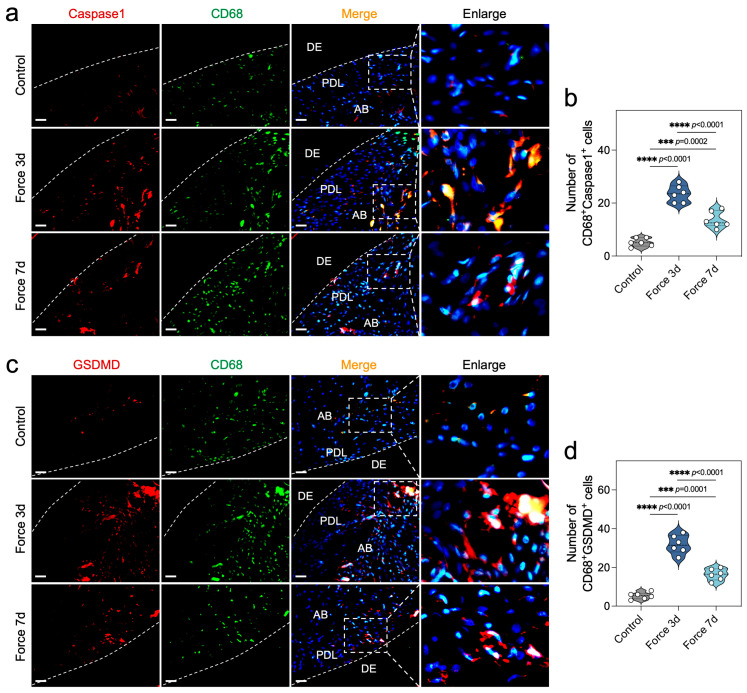
Force induces macrophage pyroptosis during OTM. (**a**,**b**) Immunofluorescence double-staining images of Caspase1 and CD68, and analysis of the number of double-positive cells in the tissues on the pressure side of the first molar in OTM rats (*n* = 6). The red fluorescence represents Caspase1, the green fluorescence indicates CD68, and the blue fluorescence corresponds to DAPI. Scale bar = 50 μm. (**c**,**d**) Immunofluorescence double-staining images of GSDMD and CD68, and analysis of the number of double-positive cells in the tissues on the pressure side of the first molar in OTM rats (*n* = 6). The red fluorescence represents GSDMD, the green fluorescence indicates CD68, and the blue fluorescence corresponds to DAPI. Scale bar = 50 μm. *** *p* < 0.001; **** *p* < 0.0001. DE: Dentin, PDL: periodontal ligament, AB: alveolar bone.

To further explore the level of force-induced macrophage pyroptosis during OTM, we meticulously assessed the expression profiles of CD68, Caspase1, and GSDMD within periodontal tissues subjected to mechanical force. CD68 has been widely recognized as a robust marker to characterize macrophages in rats, while Caspase1 is a key factor in the cleavage of GSDMD during canonical pyroptosis [20]. Employing immunofluorescence double staining, we observed a marked escalation in the population of CD68^+^Caspase1^+^ and CD68^+^GSDMD^+^ cells on the compressive side of periodontal tissues, particularly at the 3-day juncture post-force application (Figure 2a–d). Further immunohistochemical analysis revealed that the cleaved active forms of Caspase1 and GSDMD, namely Cleaved caspase1 and N-GSDMD, were also highly expressed in the compressed periodontal tissues (Figure S1a–d). Collectively, these findings underscore the capacity of mechanical force to instigate Caspase1-dependent pyroptosis in macrophages, a response that peaks at the 3-day mark, thereby shedding light on the intricate interplay between mechanical force and cellular demise in the context of OTM.

### 2.3. Force Induces Macrophage Pyroptosis and Sterile Inflammation In Vitro

**Figure 3:**
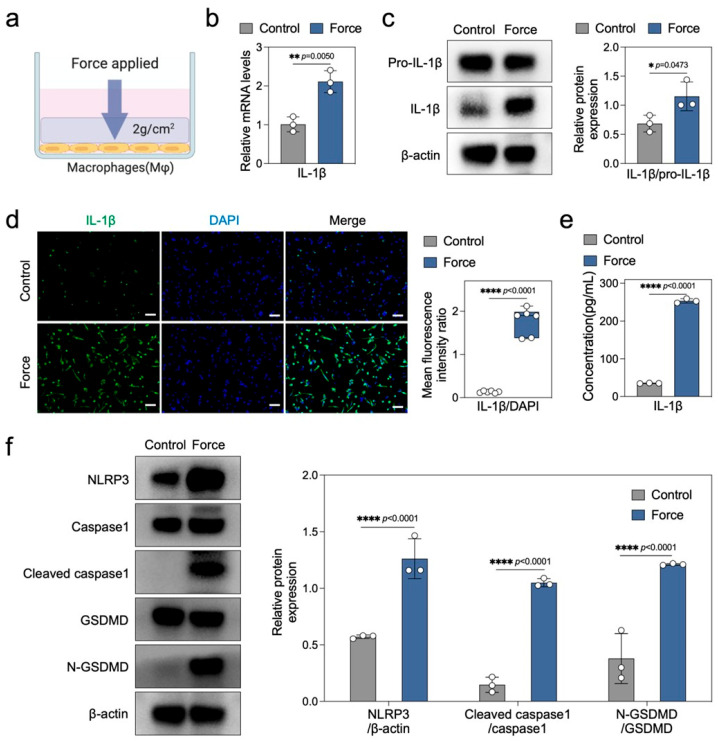
Force induces macrophage pyroptosis and sterile inflammation in vitro. (**a**) Schematic diagram of the force application model in THP-1 derived macrophages. (**b**) qPCR analysis of IL-1β (*n* = 3). (**c**) Western blotting and quantitative analysis of Pro-IL-1β and IL-1β (*n* = 3). (**d**) Immunofluorescence staining and semi-quantitative analysis of IL-1β (*n* = 6). Scale bar = 100 μm. (**e**) ELISA analysis of IL-1β in cell culture supernatants (*n* = 3). (**f**) Western blotting and quantitative analysis of NLRP3, Caspase1, Cleaved caspase1, GSDMD, and N-GSDMD (*n* = 3). * *p* < 0.05; ** *p* < 0.01; **** *p* < 0.0001.

In vitro, we employed a classic compression force loading model to explore the direct effects of mechanical force on macrophage pyroptosis and sterile inflammation (Figure 3a). Initially, we analyzed the expression of inflammation-related markers in both control and force-loaded macrophages. Real-time PCR data revealed that force significantly upregulated the mRNA expression of IL-1β, iNOS, and TNF-α (Figure 3b and Figure S2a). Western blotting showed a marked increase in the protein expression ratio of mature IL-1β to its precursor Pro-IL-1β in force-loaded macrophages (Figure 3c), corroborated by similar trends observed in IL-1β immunofluorescence and ELISA assays (Figure 3d,e). The upregulation of iNOS protein expression in force-loaded macrophages further substantiated the activation of sterile inflammation by mechanical force (Figure S2b). Subsequently, we evaluated the protein expression of pyroptosis-related markers. Western blotting results demonstrated a significant elevation in the protein levels of the NLRP3 inflammasome, Cleaved caspase1/Caspase1, and N-GSDMD/GSDMD in force-loaded macrophages (Figure 3f). Collectively, these findings indicate that mechanical force induces caspase1-dependent macrophage pyroptosis and enhances the expression and release of inflammatory factors such as IL-1β.

### 2.4. Force Disrupts Macrophage Energy Metabolism

**Figure 4:**
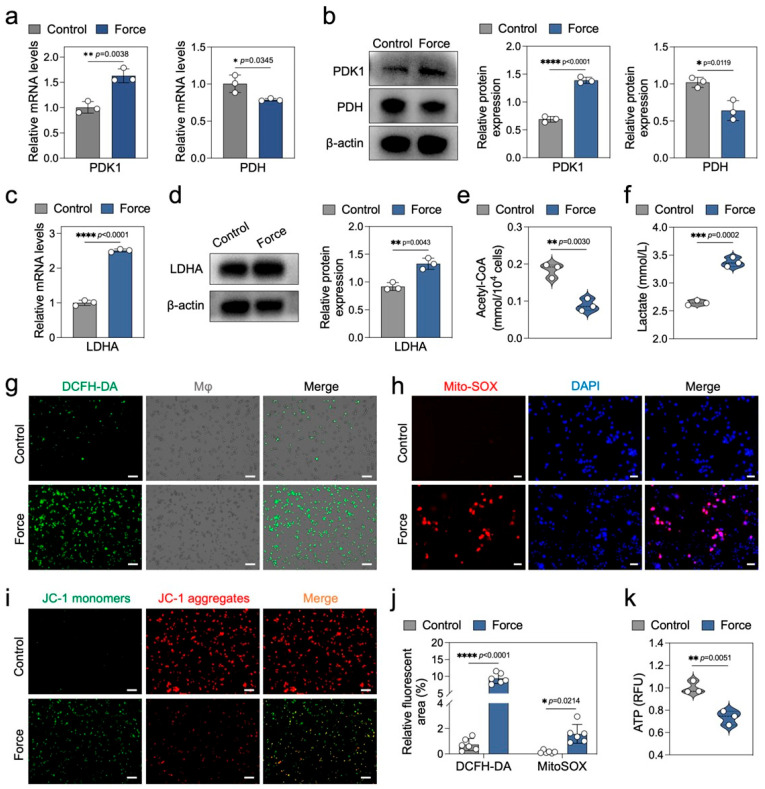
Force disrupts macrophage energy metabolism. (**a**,**b**) qPCR and Western blotting analysis of PDK1 and PDH (*n* = 3). (**c**,**d**) qPCR and Western blotting analysis of LDHA (*n* = 3). (**e**) Detection results of intracellular acetyl-CoA content (*n* = 3). (**f**) Detection results of lactate content in cell culture supernatants (*n* = 3). (**g**) Analysis of total ROS levels in cells. Scale bar = 50 μm. (**h**) Analysis of mitochondrial ROS levels. Scale bar = 50 μm. (**i**) JC-1 mitochondrial membrane potential analysis, where the ratio of red to green fluorescence intensity reflects the high or low mitochondrial membrane potential. Scale bar = 50 μm. (**j**) Semi-quantitative fluorescence analysis of DCFH-DA and MitoSOX (*n* = 6). (**k**) Analysis of intracellular ATP levels (*n* = 3). * *p* < 0.05; ** *p* < 0.01; *** *p* < 0.001; **** *p* < 0.0001.

During pyroptosis, cells may undergo metabolic reprogramming to meet the energy demands of the inflammatory response [21]. Lactate dehydrogenase A (LDHA) and pyruvate dehydrogenase (PDH) are pivotal enzymes in the glycolytic and oxidative phosphorylation pathways, respectively. LDHA catalyzes the conversion of pyruvate to lactate, while PDH converts pyruvate into acetyl-CoA, which enters the citric acid cycle [22]. The activity of PDH is negatively regulated by pyruvate dehydrogenase kinase 1 (PDK1) [23]. In this study, we observed that force upregulated PDK1 and downregulated PDH expression in macrophages at both mRNA and protein levels, suggesting inhibition of the oxidative phosphorylation pathway (Figure 4a,b). Conversely, force significantly enhanced LDHA expression, indicating activation of the glycolytic pathway (Figure 4c,d). The decrease in acetyl-CoA and increase in lactate in force-loaded macrophages further support these findings (Figure 4e,f). Additionally, force induced upregulation of the glycolytic enzyme hexokinase 2 (HK2) and downregulation of the citric acid cycle enzyme succinate dehydrogenase B (SDHB) (Figure S3a,b). Collectively, these results suggest that force disrupts pyruvate metabolism in macrophages, shifting cellular metabolism from oxidative phosphorylation to glycolysis.

Mitochondria are central to cellular energy metabolism, and their dysfunction can influence inflammasome activation and pyroptosis [24]. Reactive oxygen species (ROS) and mitochondrial membrane potential are key indicators of mitochondrial function [25]. Using DCFH-DA and Mito-SOX staining, we found that force significantly increased both total and mitochondria-specific ROS in macrophages (Figure 4g,h,j). JC-1 staining revealed a marked decrease in mitochondrial membrane potential in force-loaded macrophages (Figure 4i), indicating mitochondrial dysfunction. The NAD^+^/NADH ratio and ATP levels are also crucial markers of mitochondrial function and energy metabolic balance [26]. In this study, force significantly downregulated the NAD^+^/NADH ratio and ATP levels in macrophages (Figure 4k and Figure S3c), further highlighting the disruption of cellular energy metabolism. Overall, these findings demonstrate that force disrupts macrophage energy metabolism, primarily through the imbalance of LDHA and PDH, as well as mitochondrial dysfunction.

### 2.5. Inhibition of PDK1 Rectifies the Energy Metabolism Disorders in Force-Loaded Macrophages

**Figure 5:**
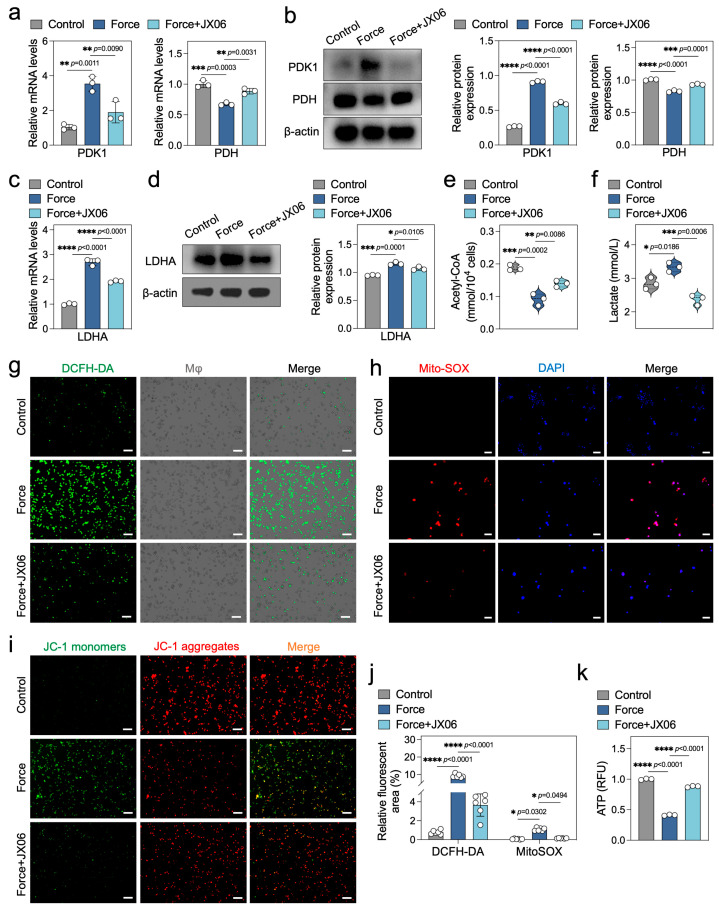
Inhibition of PDK1 rectifies the energy metabolism disorders in force-loaded macrophages. (**a**,**b**) qPCR and Western blotting analysis of PDK1 and PDH (*n* = 3). (**c**,**d**) qPCR and Western blotting analysis of LDHA (*n* = 3). (**e**) Detection results of intracellular acetyl-CoA content (*n* = 3). (**f**) Detection results of lactate content in cell culture supernatants (*n* = 3). (**g**) Analysis of total ROS levels in cells. Scale bar = 50 μm. (**h**) Analysis of mitochondrial ROS levels. Scale bar = 50 μm. (**i**) JC-1 mitochondrial membrane potential analysis, where the ratio of red to green fluorescence intensity reflects the high or low mitochondrial membrane potential. Scale bar = 50 μm. (**j**) Semi-quantitative fluorescence analysis of DCFH-DA and MitoSOX (*n* = 6). (**k**) Analysis of intracellular ATP levels (*n* = 3). * *p* < 0.05; ** *p* < 0.01; *** *p* < 0.001; **** *p* < 0.0001.

Previous studies have highlighted PDK1 as a pivotal regulatory enzyme in the metabolic transition from oxidative phosphorylation to glycolysis [27]. This transition is facilitated by PDK1 through the phosphorylation of PDH, rendering it inactive, which in turn inhibits oxidative phosphorylation and promotes glycolysis [28]. The immunohistochemical results of the OTM rat maxillary samples showed that the expression of PDK1 in the periodontal tissues under compressive force at 3 and 7 days was significantly increased, suggesting that PDK1 may be a key metabolic enzyme involved in the OTM process (Figure S4a,b). To further elucidate the critical role of PDK1 in force-induced disruptions of macrophage energy metabolism in vitro, we utilized the pharmacological inhibitor JX06 to block PDK1 activity. Our results demonstrated that JX06 effectively reversed the elevated PDK1 and diminished PDH expression in force-loaded macrophages (Figure 5a,b), and it also mitigated the increased LDHA levels to a significant extent (Figure 5c,d). As anticipated, the levels of acetyl-CoA and lactate in force-loaded macrophages were corrected following JX06 treatment (Figure 5e,f). Additionally, JX06 rescued the expression of the glycolytic key enzyme HK2 and the oxidative phosphorylation enzyme SDHB (Figure S5a,b). These findings suggest that inhibiting PDK1 with JX06 can improve the metabolic imbalance of glycolysis and oxidative phosphorylation induced by force in macrophages.

Next, we sought to determine whether inhibiting PDK1 could also rescue impaired mitochondrial function. Our results showed that JX06 significantly reduced the levels of total ROS and mitochondrial ROS in force-loaded macrophages (Figure 5g,h,j), and partially restored the decreased mitochondrial membrane potential (Figure 5i). Moreover, JX06 corrected the downregulated NAD^+^/NADH ratio and ATP levels induced by force, indicating a recovery of macrophage energy metabolism (Figure 5k and Figure S5c). In summary, inhibiting PDK1 with JX06 can effectively rectify the energy metabolism disorders induced by force in macrophages.

### 2.6. Inhibiting PDK1 Alleviates Force-Induced Macrophage Pyroptosis and Sterile Inflammation

**Figure 6:**
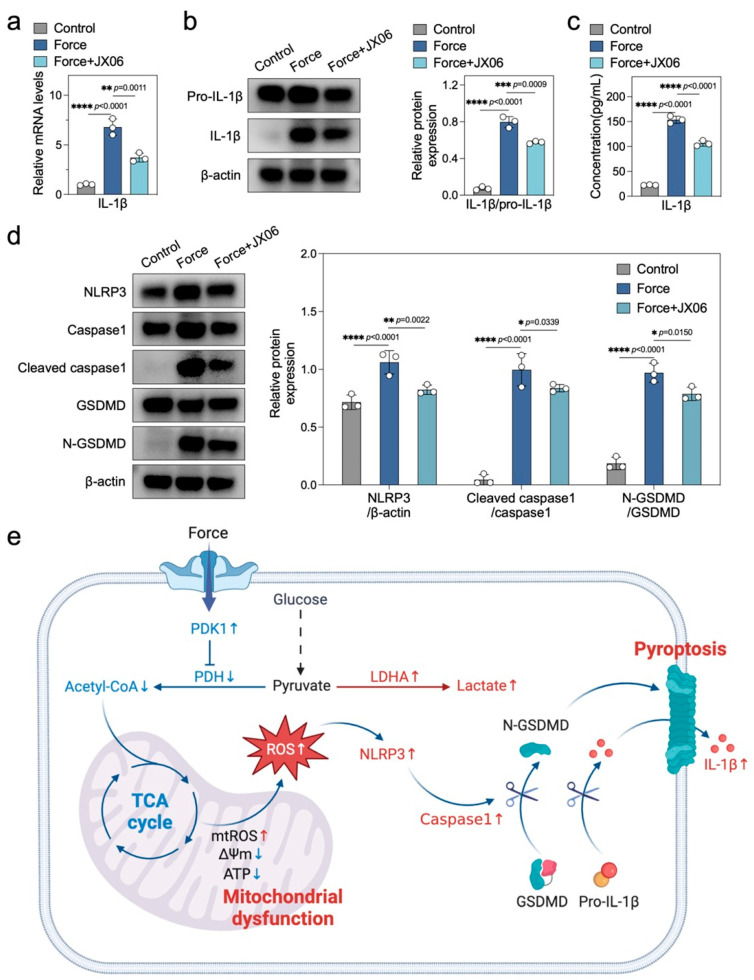
Inhibiting PDK1 alleviates force-induced macrophage pyroptosis and sterile inflammation. (**a**) qPCR analysis of IL-1β (*n* = 3). (**b**) Western blotting and quantitative analysis of Pro-IL-1β and IL-1β (*n* = 3). (**c**) ELISA analysis of IL-1β in cell culture supernatants (*n* = 3). (**d**) Western blotting and quantitative analysis of NLRP3, Caspase1, Cleaved caspase1, GSDMD, and N-GSDMD (*n* = 3). (**e**) Schematic diagram illustrating the mechanism by which force triggers macrophage pyroptosis and sterile inflammation by disrupting cellular energy metabolism. ↑ indicates upregulation, ↓ indicates downregulation. * *p* < 0.05; ** *p* < 0.01; *** *p* < 0.001; **** *p* < 0.0001.

Given that alterations in energy metabolism influence the processes of pyroptosis and inflammatory responses, and considering PDK1 as a pivotal enzyme that governs cellular energy metabolism, we propose that inhibiting PDK1 may alleviate pyroptosis and sterile inflammatory responses in force-loaded macrophages. Our research findings demonstrate that the PDK1 inhibitor JX06 significantly reduced the elevated expression of IL-1β induced by force, evident at the mRNA, protein, and secretion levels (Figure 6a–c). Furthermore, the downregulation of iNOS levels further underscores JX06′s mitigating effect on force-induced sterile inflammation (Figure S6a,b). In terms of pyroptosis markers, JX06 markedly decreased the protein levels of the inflammasome NLRP3, Cleaved caspase1/Caspase1, and N-GSDMD/GSDMD, which were upregulated in force-loaded macrophages, indicating a reduction in pyroptosis (Figure 6d). Collectively, our results suggest that inhibiting PDK1 can effectively alleviate pyroptosis and sterile inflammatory responses in macrophages subjected to force.

## 3. Discussion

In this study, we elucidated a novel mechanism by which mechanical force during OTM induces pyroptosis and sterile inflammation through the regulation of macrophage energy metabolism. Figure 6e summarizes this mechanism. Our findings establish the molecular link between mechanical force, energy metabolism, and macrophage pyroptosis, offering a new theoretical basis and potential intervention targets for the precise regulation of OTM.

Pyroptosis, a caspase1-dependent inflammatory programmed cell death, is implicated in diseases like Alzheimer’s, arthritis, and myocarditis [29,30,31], and has recently been shown to play a significant role in OTM. Studies have demonstrated that mechanical force can induce caspase-1-dependent pyroptosis in periodontal ligament progenitor cells, regulating alveolar bone remodeling during OTM [10]. Another study found that excessive orthodontic force induces pyroptosis in periodontal ligament cells, leading to root resorption [32]. In this study, we observed a significant increase in CD68^+^Caspase1^+^ and CD68^+^GSDMD^+^ cells in compressed periodontal tissue during OTM, providing the first evidence that mechanical force can induce macrophage pyroptosis during OTM, thus expanding the scope of fundamental OTM research.

Macrophages are highly sensitive to mechanical signals and can be activated by mechanical force to initiate strong pro-inflammatory responses [33,34,35]. In this study, we found that mechanical force significantly induced high expression of pro-inflammatory factors such as IL-1β, iNOS, and TNF-α in macrophages, and upregulated pyroptosis-related markers (NLRP3, Cleaved Caspase1/Caspase1, and N-GSDMD/GSDMD). Although the specific mechanisms by which mechanical force drives macrophage pyroptosis remain unclear, existing research suggests a significant link between cellular energy metabolism and pyroptosis-induced inflammatory responses. For instance, inhibiting glycolysis in alveolar macrophages can effectively reduce pyroptosis levels, thereby alleviating airway inflammation [36]. In this study, we found that mechanical force upregulated HK2, LDHA, and lactate while downregulating PDH, SDHB, and acetyl-CoA. This indicates that mechanical force enhances glycolysis and suppresses oxidative phosphorylation in macrophages. Additionally, mitochondria play a crucial role in maintaining metabolic homeostasis [37]. Studies have shown mitochondrial dysfunction can impair oxidative phosphorylation and shift cellular energy metabolism toward glycolysis [38,39,40]. Consistent with this, our results demonstrate that mechanical force not only disrupts the balance between glycolysis and oxidative phosphorylation but also significantly impairs mitochondrial function, as evidenced by reduced mitochondrial membrane potential, elevated reactive oxygen species levels, and decreased ATP synthesis. Overall, these in vitro findings on macrophage pyroptosis, inflammation, and metabolism provide new insights into the role of energy metabolism in mechanical force-related inflammatory pathologies, although the complex regulatory networks involved require further investigation.

PDK1, a key regulator of glycolysis and oxidative phosphorylation [28], plays a central role in mechanical force-induced metabolic remodeling in macrophages. In this study, we found that mechanical force significantly upregulated PDK1 expression, thereby inhibiting PDH activity and preventing pyruvate from entering the TCA cycle, ultimately shifting energy metabolism toward glycolysis. Several studies have shown that inhibiting PDK1 can promote pyruvate entry into mitochondria for oxidative phosphorylation, making it a potential strategy to rescue energy metabolism crises [17,41,42]. Based on this, we used the PDK1-specific inhibitor JX06 for intervention. We found that inhibiting PDK1 restored oxidative phosphorylation and improved mitochondrial function, suggesting energy metabolism imbalance may be a key driver of mechanical force-induced pyroptosis and sterile inflammation. Furthermore, studies have reported that in acute lung injury models, inhibiting glycolysis downregulates pyroptosis-related gene expression [36], while improving mitochondrial function also helps mitigate pyroptosis [43]. Our study confirms that inhibiting PDK1 not only corrects energy metabolism disorders but also effectively suppresses pyroptosis and sterile inflammation. These findings highlight PDK1′s critical role in mechanical force-triggered macrophage pyroptosis and sterile inflammation, providing a new theoretical foundation and potential intervention target for OTM inflammation management.

Several limitations of our study warrant further investigation. First, while we investigated sterile inflammation and pyroptosis marker expression in OTM tissues, further work is needed to explore energy metabolism-related marker expression. Second, metabolic regulation involves complex biochemical pathways, and future studies should incorporate metabolomic analyses to systematically dissect mechanical force-induced changes in energy metabolism. Finally, the barriers to applying PDK1 in OTM models need to be addressed, such as developing engineered drug delivery systems. We look forward to exploring and resolving these issues in future research.

## 4. Materials and Methods

### 4.1. Animals and Orthodontic Force Treatment

The animal experiments involved in this study were approved by the Ethics Committee of the College of Stomatology, Chongqing Medical University (license number: CQHS-REC-2024 (LSNo. 172)). Healthy male Sprague Dawley rats (6–8 weeks old) of clean grade were selected and randomly divided into three groups: the control group, the force 3d group, and the force 7d group, with *n* = 6 rats per group.

As described in previous studies [10], mechanical force was applied to the rats. In brief, under general anesthesia, an orthodontic nickel–titanium spring was used to connect the first maxillary molar and the incisor of the rat, applying a continuous orthodontic force of approximately 50 g to simulate the clinical process of tooth movement. The experimental period was 7 days, during which the rats’ conditions were observed daily, and the force application devices were checked for detachment to ensure the smooth progress of the experiment.

### 4.2. Micro-CT Scanning and Measurement of Tooth Movement Distance

The rats were sacrificed on day 3 and day 7 after the application of orthodontic force, and their maxillae were isolated and fixed. The maxillae were subjected to multi-layered scanning using a Micro-CT scanner (SCANCO Medical AG, Brüttisellen, Switzerland). The scanning was performed at a voltage of 80 kV, a current of 100 μA, and a resolution of 50 μm. After the scanning was completed, three-dimensional image data of the rat maxillae were obtained. The distance of tooth movement was assessed by measuring the distance from the distal surface of the first molar to the mesial surface of the second molar on the reconstructed images of the maxilla. An investigator who was blinded to the group allocation measured each sample three times and took the average to obtain the tooth movement distance for that sample.

### 4.3. Hematoxylin and Eosin (H&E) Staining

After fixation of the rat maxillary tissues with 4% paraformaldehyde (Biosharp, Hefei, China), decalcification and dehydration processes were carried out, followed by routine paraffin embedding of the tissue samples. These tissues were then cut into 5 μm thick paraffin sections, which were subsequently dewaxed with xylene, dehydrated through an ethanol gradient, and then stained using an H&E staining kit (Solarbio, Beijing, China). The specific steps were as follows: hematoxylin staining for 10 min, eosin staining for 5 min, followed by dehydration, clearing, and mounting. The stained sections were observed under a microscope to document the morphological changes in the tooth roots and periodontal tissues.

### 4.4. Tartrate-Resistant Acid Phosphatase (TRAP) Staining

The TRAP staining working solution was prepared according to the instructions of the TRAP staining kit (Solarbio, Beijing, China) to ensure sufficient quantity. After dewaxing and rehydrating the paraffin sections, the TRAP staining working solution was added dropwise and incubated in the dark for 30 min, followed by gentle rinsing with distilled water to remove any unbound staining solution. Sections were counterstained with hematoxylin and then mounted. TRAP-positive cells (appearing red) were observed under a microscope, and images were captured to analyze the number and distribution of osteoclasts in the periodontal tissues.

### 4.5. Immunohistochemical Staining

Antigens on the dewaxed and rehydrated paraffin sections were retrieved using microwave heating. The sections were incubated overnight at 4 °C in the dark with a diluted primary antibody against IL-1β (1:100, Bioss, Beijing, China), Caspase1 P20 (1:50, Santa Cruz, Dallas, TX, USA), GSDMD-N (1:50, Immunoway, Newark, DE, USA), and PDK1 (1:50, Proteintech, Wuhan, China). The following day, the sections were incubated for 1 h at room temperature with the corresponding secondary antibody (1:200, Beyotime, Shanghai, China). After incubation with the secondary antibody, the sections were stained with DAB chromogen solution (ZSGB-BIO, Beijing, China) until a distinct brown-yellow positive signal appeared. Finally, the sections were dehydrated, cleared, and then mounted. The immunohistochemical staining results were observed and recorded under a microscope to analyze the expression and localization of the target protein IL-1β in the periodontal tissues.

### 4.6. Immunofluorescence Staining of Tissues

The maxillary samples from the control group and the OTM groups were sectioned in the same orientation. Paraffin sections were labeled with primary antibodies against CD68 (Affinity, Cincinnati, OH, USA), Caspase1 (Santa Cruz, Dallas, TX, USA), and GSDMD (Santa Cruz, Dallas, TX, USA). The sections were then incubated with CoraLite488/594-conjugated secondary antibodies (Proteintech, Wuhan, China). Nuclei were stained with DAPI (Beyotime, Shanghai, China) and the sections were mounted. Under an inverted fluorescence microscope, the maxillary first molar periodontal tissues were selected as the region of interest (ROI). The positive expression of CD68 labeled with red fluorescence and Caspase1 and GSDMD labeled with green fluorescence were observed within the ROI, and the number of double-positive cells for CD68⁺Caspase1⁺ and CD68⁺GSDMD⁺ was counted.

### 4.7. Force Loading and Treatments on Macrophages In Vitro

Human THP-1 monocytes (American Type Culture Collection, ATCC, Manassas, VA, USA) were cultured in RPMI-1640 complete medium (Pricella, Wuhan, China). Approximately 5 × 10^5^ THP-1 cells were seeded in standard 12-well plates and treated with PMA (Phorbol 12-myristate 13-acetate, 100 ng/mL, MCE, Monmouth Junction, NJ, USA) for 24 h to induce their differentiation into adherent macrophages. According to the established and published method for simulating orthodontic compressive force [10,35,44], a specially customized glass plate was gently loaded onto the cell surface to generate a compressive force of 2 g/cm^2^, which was maintained for 24 h before collecting the cells or supernatant for further experiments. In some experiments, the macrophages were pre-treated with the PDK1 inhibitor JX06 (10 μM, MCE, Monmouth Junction, NJ, USA) for 1 h before force application.

### 4.8. Quantitative Reverse Transcription-PCR (RT-qPCR)

Total RNA from macrophages was extracted using Trizol reagent (Takara, Otsu, Shiga, Japan), and 500 ng of the extracted RNA was reverse-transcribed into cDNA using a reverse transcription kit (Takara, Otsu, Shiga, Japan). Specific primers were designed to amplify target genes such as IL-1β, iNOS, TNF-α, PDK1, PDH, LDHA, HK2, and SDHB. The reaction system included cDNA template, primers, SYBR Green dye (Takara, Otsu, Shiga, Japan), and reaction buffer. β-actin was used as the internal reference gene, and the relative expression of the target genes was calculated using the 2^−ΔΔCt^ method. The sequences of the primers (Sangon Biotech, Shanghai, China) are listed in Table S1.

### 4.9. Western Blotting Analysis

After extracting total protein from macrophages, the protein concentration was measured. Protein samples (20 μg per group) were separated by SDS-PAGE electrophoresis and then transferred onto PVDF membranes. The PVDF membranes were incubated overnight at 4 °C with specific primary antibodies (such as anti-IL-1β, anti-iNOS, anti-NLRP3, anti-Caspase1, anti-Cleaved caspase1, anti-GSDMD, anti-N-GSDMD, anti-PDK1, anti-PDH, anti-LDHA, anti-HK2, anti-SDHB, and anti-β-actin, diluted at 1:1000). A complete list of antibodies is provided in Table S2. On the second day, the membranes were incubated for 1 h with HRP-conjugated secondary antibodies (diluted at 1:5000). Finally, the membranes were developed using ECL chemiluminescent reagent (Beyotime, Shanghai, China), exposed, and the results were recorded. The relative expression levels of the target proteins were analyzed using ImageJ software (version 2.3.0).

### 4.10. Immunofluorescence Staining of Cells

After fixation with 4% paraformaldehyde (Biosharp, Hefei, China) and permeabilization with 0.1% Triton X-100 (Beyotime, Shanghai, China), the treated macrophages were blocked with 5% fetal bovine serum (Lonsera, Montevideo, Uruguay) for 30 min. Subsequently, the cells were incubated with a specific primary antibody (anti-IL-1β, diluted at 1:200) overnight at 4 °C. On the second day, CoraLite48-conjugated secondary antibody was added and incubated for 1 h at room temperature. Finally, nuclei were stained with DAPI (Beyotime, Shanghai, China). The cell staining was observed under a fluorescence microscope, and images were captured to document the expression and distribution of the target protein within the cells.

### 4.11. Enzyme Linked Immunosorbent Assay (ELISA)

The supernatants of treated macrophage cultures were collected by centrifugation. The concentration of IL-1β in the supernatants was measured according to the protocol of the IL-1β ELISA kit (Servicebio, Wuhan, China). In brief, specific antibodies and enzyme conjugates were added to the samples in the ELISA plate. After the colorimetric reaction, the absorbance of the reaction products in each well was measured using a multifunctional microplate reader. The concentration of the IL-1β in the samples was calculated based on a standard curve.

### 4.12. Acetyl-CoA and Lactate Content Detection

Macrophage lysates and their supernatants were collected. The acetyl-CoA content in the cell lysates was measured using the Acetyl-CoA Assay Kit (Solarbio, Beijing, China) using the colorimetric method. The lactate content in the supernatant samples was measured using the Lactate Assay Kit (NJJCBIO, Nanjing, China) using the colorimetric method. The concentrations of acetyl-CoA and lactate were calculated based on specific formulas.

### 4.13. ROS Detection

The levels of total ROS and mitochondrial-specific ROS in macrophages were detected using the DCFH-DA fluorescent probe (Beyotime, Shanghai, China) and the Mito-SOX Red fluorescent probe (Beyotime, Shanghai, China), respectively. The cells were incubated with DCFH-DA or Mito-SOX Red for 30 min, and then excess dye was washed away with PBS. Fluorescence intensity was observed under a fluorescence microscope to analyze the levels of ROS.

### 4.14. Mitochondrial Membrane Potential Measurement

Mitochondrial membrane potential was detected using the JC-1 fluorescent probe (Beyotime, Shanghai, China). Cells were incubated with JC-1 staining solution for 30 min and then washed with PBS. The red and green fluorescence intensities of JC-1 were observed under a fluorescence microscope, and changes in mitochondrial membrane potential were determined by analyzing the red-to-green fluorescence ratio.

### 4.15. ATP Level Determination

The intracellular ATP content of treated macrophages was measured using the ATP Assay Kit (Beyotime, Shanghai, China). Briefly, after lysing the cell samples, the working solution was added and allowed to react fully. The RLU (relative light units) values of the standards and samples were measured using a luminometer, and the ATP concentration in each sample was calculated based on the absorbance of the standards.

### 4.16. NAD^+^ and NADH Detection

The macrophage lysate was collected and processed according to the kit’s instructions (Beyotime, Shanghai, China). The content of NAD^+^ and NADH in the samples was determined using the colorimetric method. The NAD^+^/NADH ratio was calculated to analyze the redox state of the cells.

### 4.17. Statistical Analysis

Data analysis was performed using GraphPad Prism software (version 10.2.3). Data are expressed as mean ± standard deviation (mean ± SD). For comparisons between groups, *t*-tests or one-way analysis of variance (ANOVA) was used, with *p* < 0.05 considered statistically significant. Bar charts, box plots, violin plots, and other graphical representations were created based on experimental data to visually display the results.

## 5. Conclusions

In summary, our findings demonstrate that mechanical force induces macrophage pyroptosis and sterile inflammation, with the underlying mechanisms involving metabolic imbalances characterized by disrupted ratios of LDHA to PDH and mitochondrial dysfunction. We further show that inhibiting PDK1 effectively restores this metabolic balance, thereby attenuating force-induced macrophage pyroptosis and sterile inflammation. Collectively, these results offer new insights into the precise and efficient modulation of sterile inflammatory processes during OTM and identify PDK1 as a potential therapeutic target.

## Data Availability

The original contributions presented in this study are included in the article/Supplementary Materials. Further inquiries can be directed to the corresponding authors.

## Supplementary Materials

The supporting information can be downloaded at https://www.mdpi.com/article/10.3390/ijms26073321/s1.
