# Supplementary material for: Mechanical Force Triggers Macrophage Pyroptosis and Sterile Inflammation by Disrupting Cellular Energy Metabolism
Source: Int J Mol Sci. 2025 Apr 2;26(7):3321. doi: 10.3390/ijms26073321 (PMC11989687; doi:10.3390/ijms26073321)
Supplement: Supplementary file 1 [file ijms-26-03321-s001.zip › ijms-3511237-supplementary.pdf]

Mechanical Force Triggers Macrophage Pyroptosis and Sterile Inflammation by Disrupting Cellular Energy Metabolism

Hao Tan [1,2,3,†], Guoyin Yang [1,2,3,†], Ye Zhu [1,2,3], Xinyi He [1,2,3], Lan Yang [1,2,3], Yun Hu [1,2,3,*] and Leilei Zheng [1,2,3,*]

[1] College of Stomatology, Chongqing Medical University, Chongqing 401147, China;
18375761068@163.com (H.T.); 2022120706@stu.cqmu.edu.cn (G.Y.);
zhuye0617@outlook.com (Y.Z.); 2020121237@stu.cqmu.edu.cn (X.H.);
2021440057@stu.cqmu.edu.cn (L.Y.)

[2] Chongqing Key Laboratory of Oral Diseases and Biomedical Sciences, Chongqing 401147, China

[3] Chongqing Municipal Key Laboratory of Oral Biomedical Engineering of Higher Education, Chongqing 401147, China

[*] Correspondence: 500188@hospital.cqmu.edu.cn (Y.H.); zhengleileicqmu@hospital.cqmu.edu.cn (L.Z.)

[†] These authors contributed equally to this work.

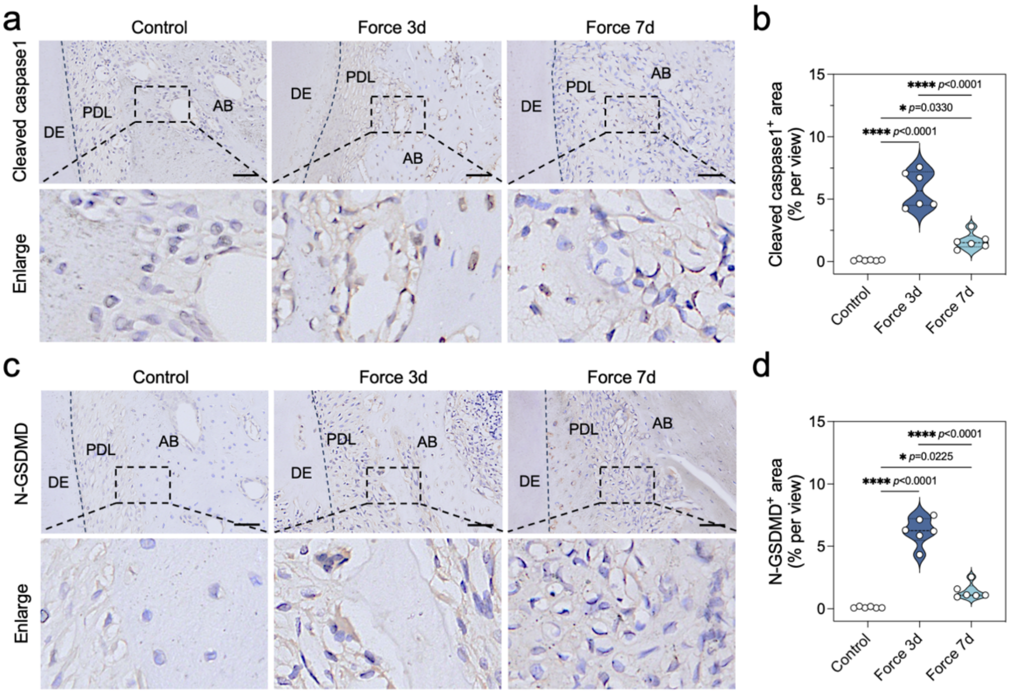

Figure S1. (**a, b**) Immunohistochemical staining of Cleaved caspase1 and analysis of the proportion of positive areas in tissues on the pressure side of the first molar in OTM rats (n = 6). Scale bar = 50 µm. (**c, d**) Immunohistochemical staining of N-GSDMD and analysis of the proportion of positive areas in tissues on the pressure side of the first molar in OTM rats (n = 6). Scale bar = 50 µm. *$p < 0.05$; ****$p < 0.0001$. DE: Dentin, PDL: Periodontal ligament, AB: Alveolar bone.

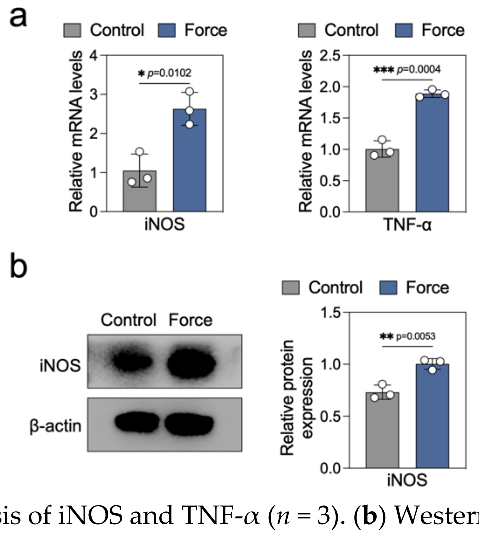

Figure S2. (**a**) qPCR analysis of iNOS and TNF-α (n = 3). (**b**) Western Blotting and quantitative analysis of iNOS (n = 3). *$p < 0.05$; **$p < 0.01$; ***$p < 0.001$.

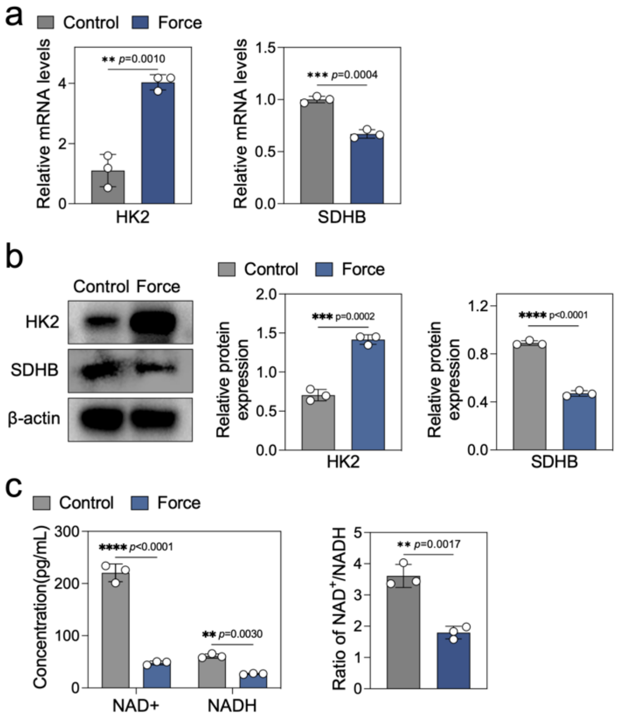

Figure S3. (**a**) qPCR analysis of HK2 and SDHB (n = 3). (**b**) Western Blotting and quantitative analysis of HK2 and SDHB (n = 3). (**c**) The concentrations of NAD^+ and NADH, as well as the NAD^+/NADH ratio in macrophages (n = 3). **$p < 0.01$; ***$p < 0.001$; ****$p < 0.0001$.

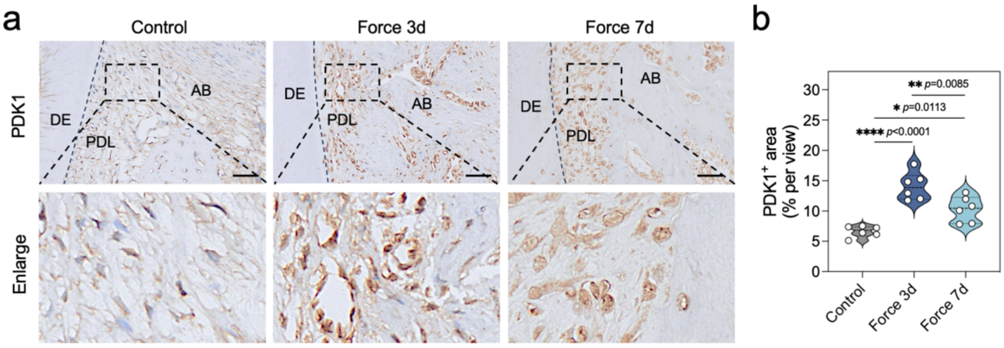

Figure S4. Immunohistochemical staining of PDK1 and analysis of the proportion of positive areas in tissues on the pressure side of the first molar in OTM rats (n = 6). Scale bar = 50 μm. *$p < 0.05$; **$p < 0.01$; ****$p < 0.0001$. DE: Dentin, PDL: Periodontal ligament, AB: Alveolar bone.

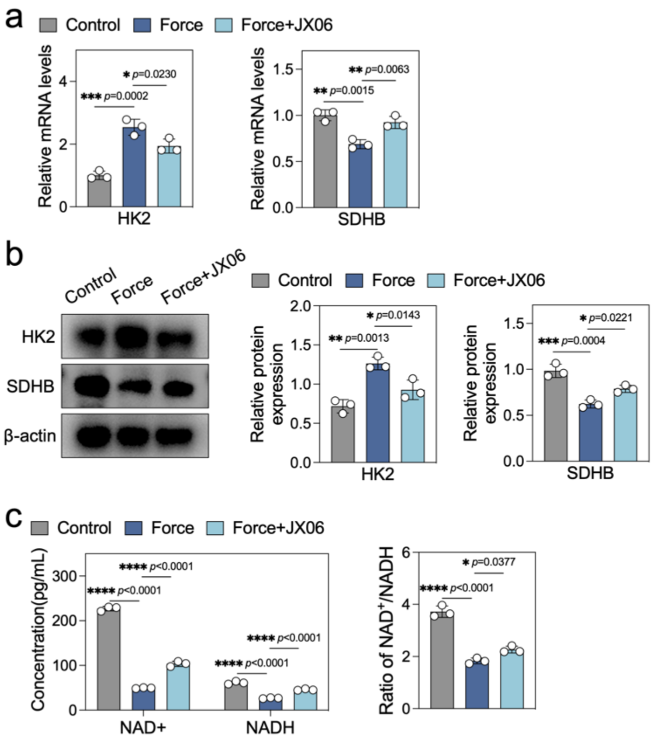

Figure S5. (**a**) qPCR analysis of HK2 and SDHB ($n = 3$). (**b**) Western Blotting and quantitative analysis of HK2 and SDHB ($n = 3$). (**c**) The concentrations of NAD^+ and NADH, as well as the NAD^+/NADH ratio in macrophages ($n = 3$). $^*p < 0.05$; $^{**}p < 0.01$; $^{***}p < 0.001$; $^{****}p < 0.0001$.

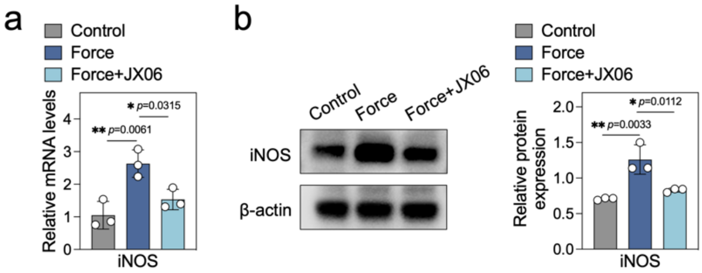

Figure S6. (**a**) qPCR analysis of iNOS ($n = 3$). (**b**) Western Blotting and quantitative analysis of iNOS ($n = 3$). $^*p < 0.05$; $^{**}p < 0.01$.

Table S1. Primers for Quantitative Reverse Transcription-PCR.

Gene	Forward primer (5′ to 3′)	Reverse primer (5′ to 3′)
IL-1β	GCCAGTGAAATGATGGCTTATT	AGGAGCACTTCATCTGTTTAGG
TNF-α	GCCCATGTTGTAGCAAACCC	CGGGTACAACATCGTTTGGG
iNOS	CGCATGACCTTGGTGTTTGG	CATAGACCTTGGGCTTGCCA
HK2	GAGCCACCACTCACCCTACT	CCAGGCATTCGGCAATGTG
LDHA	ATGGCAACTCTAAAGGATCAGC	CCAACCCCAACAACTGTAATCT

PDK1	ACCAGGACAGCCAATACAA	TACCCAGCGTGACATGAA
β-actin	TCCATCGGAGCCGAAGAAATC	GTGTCGGTGGATCAAAGCACA
PDH	GCTCATGGTCAGCAGTCA	AGCAGCCGATCCTGTTT
SDHB	TGAACATCAATGGAGGCA	GCTCAAATCGGGAACAAG

Table S2. Antibodies used in this study.

Target antigen	Vendor	Catalog #	Working concentration
IL-1β	Bioss	bs-0812R	1:100 (IHC)
Caspase-1 P20	Santa	Sc-398715	1:50 (IHC)
GSDMD-N	Immunoway	YT7991	1:50 (IHC)
CD68	Affinity	DF7518	1:200 (IF)
GSDMD	Santa	Sc-393581	1:200 (IF)
Caspase1	Santa	Sc-56036	1:200 (IF)
IL-1β	CST	12703	1:1000 (WB), 1:200 (IF)
β-actin	Proteintech	20536-1-AP	1:1000 (WB)
NLRP3	Abcam	ab263899	1:1000 (WB)
GSDMD	CST	39754	1:1000 (WB)
Caspase1	CST	3866	1:1000 (WB)
Cleaved caspase1	CST	4199	1:1000 (WB)
HK2	Proteintech	22029-1-AP	1:1000 (WB)

LDHA	Proteintech	19987-1-AP	1:1000 (WB)
PDK1	Proteintech	18262-1-AP	1:1000 (WB), 1:50 (IHC)
PDH	CST	3205	1:1000 (WB)
SDHB	Proteintech	10620-1-AP	1:1000 (WB)
iNOS	Proteintech	18985-1-AP	1:1000 (WB)
HRP Goat Anti-rabbit IgG (H+L)	Beyotime	A0208	1:5000 (WB)
HRP Goat Anti-Mouse IgG (H+L)	Beyotime	P0946	1:5000 (WB)
CoraLite488 – conjugated Goat Anti-Rabbit IgG (H+L)	Proteintech	SA00013-2	1:200 (IF)
CoraLite594 – conjugated Goat Anti-Rabbit IgG (H+L)	Proteintech	SA00013-4	1:200 (IF)
